# An outbreak of *Yersinia pseudotuberculosis* at a zoo aviary in central California: case series and field investigation

**DOI:** 10.1177/10406387261440908

**Published:** 2026-04-19

**Authors:** Raúl A. Resendiz-Pozos, Todd Cornish, Melissa Macías-Rioseco, Maria Soper, Cassandra Lapham-Simpson, Audrey Siegrist, Jennine Ochoa

**Affiliations:** California Animal Health and Food Safety Laboratory System, Tulare Branch, School of Veterinary Medicine, University of California–Davis, Tulare, CA, USA; California Animal Health and Food Safety Laboratory System, Tulare Branch, School of Veterinary Medicine, University of California–Davis, Tulare, CA, USA; California Animal Health and Food Safety Laboratory System, Tulare Branch, School of Veterinary Medicine, University of California–Davis, Tulare, CA, USA; California Animal Health and Food Safety Laboratory System, Tulare Branch, School of Veterinary Medicine, University of California–Davis, Tulare, CA, USA; Fresno Chaffee Zoo, Fresno, CA, USA; Fresno Chaffee Zoo, Fresno, CA, USA; California Animal Health and Food Safety Laboratory System, Tulare Branch, School of Veterinary Medicine, University of California–Davis, Tulare, CA, USA

**Keywords:** beautiful fruit dove, golden crested myna, hooded pitta, *Yersinia pseudotuberculosis*, yersiniosis, zoo aviary

## Abstract

Here, we detail the pathology findings in a hooded pitta (*Pitta sordida*), a beautiful fruit dove (*Ptilinopus pulchellus*), and a golden-crested myna (*Ampeliceps coronatus*), all housed together in a mixed-species aviary at a zoo in central California that experienced mortalities over 3 mo. *Yersinia pseudotuberculosis* was identified as the primary pathogen, responsible for necrotizing heterophilic and histiocytic hepatitis, splenitis, pneumonia, nephritis, myositis, myocarditis, and enteritis. We also share the results of a field investigation to identify the source(s) of *Y. pseudotuberculosis* infection in the aviary; no bacteria were detected in samples of water, soil, feces, earthworms, earwigs, or organs from mice and fox squirrels, leading to the suspicion that the hooded pitta was a carrier. The zoo’s veterinary team implemented control measures, including an 11-mo quarantine and the acquisition of birds before the cold, rainy season, allowing them to acclimate safely. No new cases of yersiniosis occurred at the aviary in the year following the outbreak.

*Yersinia pseudotuberculosis* is a gram-negative, non–spore-forming, facultative anaerobic coccobacillus found in >110 species, including birds, mammals, and reptiles.^16^ *Y. pseudotuberculosis* can be zoonotic, with human infection usually from contaminated food or water.^16^ The bacterium typically resides in the intestines of subclinically infected hosts, such as wild birds, rodents, wild boars, livestock, primates, lab animals, and insects.^5,10,16^ *Y. pseudotuberculosis* has 6 genetic groups and 21 serotypes based on the O-antigen.^5,11^

In birds, sudden death is common, following signs of lethargy, diarrhea, and reduced appetite.^11^ The pathogenicity of *Y. pseudotuberculosis* depends on host immune status, bacterial virulence, and environmental factors.^2^ *Y. pseudotuberculosis* has been reported to cause clinical disease in animals in zoos,^2,7^ primate research centers, and non-domestic ruminant farms,^5^ mainly affecting bovids, cervids,^14^ felids,^17^ bats, non-human primates,^10^ rodents,^11^ rabbits,^12^ hornbills, toucans, pigeons, and parrots.^11^ *Y. pseudotuberculosis* is also an abortigenic agent in ruminants.^3^

Three birds that died over 3 mo in the winter and early spring 2025 were autopsied at the California Animal Health and Food Safety Laboratory System (CAHFS; Tulare, CA, USA). The deceased included a 3-y-old, male hooded pitta (*Pitta sordida*); a 4-y-old, male beautiful fruit dove (*Ptilinopus pulchellus*); and a 6-y-old, female golden crested myna (*Ampeliceps coronatus*). They were housed together in a zoo aviary in central California, alongside 19 other Southeast Asian birds, and near exhibits with Malayan tigers (*Panthera tigris tigris*), Komodo dragons (*Varanus komodoensis*), and sloth bears (*Melursus ursinus*). The birds were found dead during morning checks of their exhibit. The hooded pitta had no signs of illness; the fruit dove and golden crested had respiratory distress, fluffed wings, and lethargy for 2 d. Both were hospitalized with oxygen therapy; the golden crested myna received doxycycline and fluids.

The 3 carcasses were in fair-to-good postmortem condition. The 46.7-g hooded pitta was in poor body condition with a moderately prominent keel, mild-to-moderate pectoral muscle atrophy, and scant internal fat. The 58.5-g beautiful fruit dove had similar gross findings. The 56.8-g golden crested myna was in good nutritional condition, with proper muscle and fat stores, mild dehydration, and urates in the cloaca. All had an empty crop and proventriculus, with grit and fibrous material in the ventriculus; most intestines were empty. The lungs were mildly congested.

In the hooded pitta, the liver had 1–2-mm white-or-yellow foci on the capsular and cut surfaces. The kidneys were faintly mottled. The beautiful fruit dove had 1–2-mm white nodules in the lungs and liver, with necrotic cores (**Fig. 1A**). No significant gross lesions were noted in the golden crested myna.

**Figure 1.**
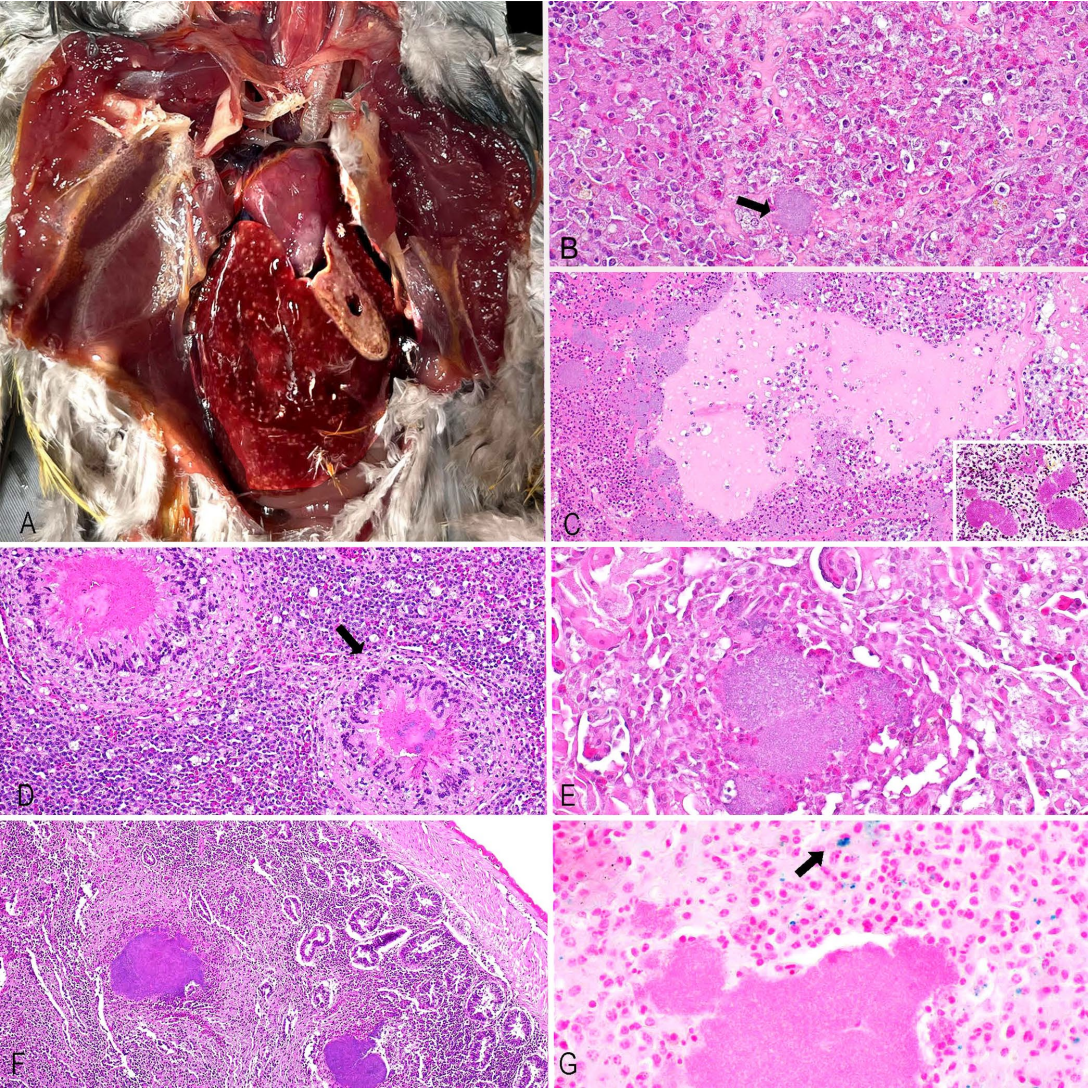
Gross and microscopic findings in an outbreak of *Yersinia pseudotuberculosis* infection at a zoo aviary in central California. **A.** Liver of the beautiful fruit dove, with well-defined, pale-tan, 1–2-mm nodules. **B.** Liver of the hooded pitta, with necrosis, heterophilic and histiocytic inflammation, and bacterial colonies (arrow). H&E. **C.** Lung from the beautiful fruit dove, with necrosis and abundant bacterial colonies. H&E. Inset: gram-negative coccobacilli. Gram stain. **D.** Spleen from the beautiful fruit dove. The central area of necrosis in granulomas is surrounded by a band of viable and degenerate heterophils, multinucleate giant cells, and an outer rim of fibrous connective tissue (arrow). H&E. **E**. Kidney from the hooded pitta. Necrosis, mixed with bacterial colonies, and the surrounding degenerate tubular epithelium. H&E. **F.** Small intestine from the golden crested myna. Mucosa and submucosa with inflammation, necrosis, and large bacterial colonies. H&E. **G.** Spleen from the beautiful fruit dove, with a few blue hemosiderin granules (arrow) near an area of necrosis. Perl stain.

Histologic examination of the liver, lung, spleen, skeletal muscle, kidney, heart, and intestine was performed. Tissues were fixed in 10% neutral-buffered formalin for 24 h, processed routinely to produce 4-μm sections, and stained with H&E, Gram/Brown–Hopps, and Perl Prussian blue. In general, the histologic findings for the 3 carcasses were similar. They included multifocal areas in which the parenchyma was replaced by well-demarcated areas of lytic necrosis mixed and surrounded by variable numbers of viable and necrotic heterophils, macrophages, multinucleate giant cells, fibrin, and large (up to 200-μm wide) amphophilic colonies of 1–2-μm gram-negative coccobacilli. Adjacent parenchyma had degeneration of variable severity (**Fig. 1B–G**). Iron was variably present in affected organs (**Table 1**).

**Table 1.** Heterophilic and histiocytic inflammation and Perl stain results from 3 birds in a *Yersinia pseudotuberculosis* outbreak at a zoo aviary in central California.

Bird	Liver	Lung	Spleen	Kidney	Skeletal muscle	Heart	Intestine
Hooded pitta	+ P+	+ P–	+ P+	+ P–	+ P–	NE	NE
Beautiful fruit dove	+ P+	+ P–	+ P+	+ P–	NE	+ P–	NE
Golden crested myna	+ P+	NE	+ P+	NE	NE	NE	+ P–

+ = inflammatory lesions; NE = not evaluated; P– = Perl negative; P+ = Perl positive.

All birds were tested for influenza A virus by PCR on pharyngeal swabs.^15^ Aerobic cultures were performed on liver, lung, and small intestine. Tissues were sampled and then inoculated onto 5% sheep blood agar (SBA; Hardy), and MacConkey agar (MAC; Hardy), streaked for isolation, and incubated at 35 ± 2°C with 5–10% CO_2_ for 24–48 h. Bacterial isolates were identified by a combination of biochemical testing of oxidase-negative, non–lactose-fermenting, clear, flat colonies with light-pink centers and matrix-assisted laser desorption/ionization–time-of-flight mass spectrometry (Bruker; **Table 2**).

**Table 2.** Culture and PCR test results, organized by bird and organs from a *Yersinia pseudotuberculosis* outbreak at a zoo aviary in central California.

Bird	Bacterial culture				Influenza A virus PCR
	Liver	Lung	Spleen	Intestine	
Hooded pitta	Y. pseudotb (mod)	ND	ND	ND	ND
Beautiful fruit dove	Y. pseudotb (lg)	Y. pseudotb (sm)	ND	ND	ND
Golden crested myna	Y. pseudotb (mod) S. gallolyticus (mod)	ND	ND	Y. pseudotb (mod)	ND

lg = isolated or detected in large numbers; mod = isolated or detected in moderate numbers; ND = not detected; sm = isolated or detected in small numbers; S. gallolyticus = *Streptococcus gallolyticus*; Y. pseudotb = *Yersina pseudotuberculosis*.

After yersiniosis was confirmed in all 3 birds, a field investigation was conducted at the zoo aviary to identify potential sources of infection for the birds, based on known common reservoirs of *Y. pseudotuberculosis* and areas with high potential for ingestion or oral exposure.^5,16^ Food storage and preparation sites were investigated and found to have extensive biosecurity controls in place. An integrated pest, rodent, and insect management program is active at this facility, with traps checked daily and bait traps serviced weekly.

We collected environmental samples (soil, water, avian feces) from the aviary and also sampled opportunistically captured earthworms (*Lumbricus terrestris*), earwigs (*Euborella annulipes)*, cloacal swabs from 6 live birds in the aviary, and 7 carcasses obtained from rodent control trapping near the aviary (2 house mice [*Mus musculus*] and 5 eastern fox squirrels [*Sciurus niger*]). Samples of water, soil, feces, earthworms, earwigs, and organ cultures of lung, liver, spleen, mesenteric lymph node, and large intestine from mice and colon and liver from fox squirrels were cultured in accordance with the standard operating procedure for cold enrichment. Samples were inoculated into MAC agar and Cefsulodin–Irgasan–Novobiocin agar (Hardy), and swabs were placed in 5 mL sterile PBS (Remel). Plates were incubated aerobically at 24–26°C for 42–48 h and observed for typical colonies at 18–24 and 42–48 h. Swabs were held in PBS at 3–5°C for 3 wk and were subcultured weekly.

In all 3 cases, *Y. pseudotuberculosis* was considered the primary pathogen, causing necrotizing heterophilic and histiocytic hepatitis (3), splenitis (3), pneumonia (2), nephritis (2), myositis (1), myocarditis (1), and enteritis (1; Table 1). Serotyping or genotyping of *Y. pseudotuberculosis* isolates was not performed in our case series. In the golden crested myna, *Streptococcus gallolyticus*—an emerging avian pathogen^6^—was also isolated from the liver (Table 2); this was likely an opportunistic infection that may have contributed to death. *Yersinia enterocolitica* was isolated from the small intestine of the squirrels, likely an indication of their carrier status.^1^ The remaining environmental and rodent samples were negative for *Y. pseudotuberculosis*.

A previous report from CAHFS indicated that avian *Y. pseudotuberculosis* is endemic in captive bird species in California.^5,16^ Typical lesions reported in avian cases include hepatitis, splenitis, nephritis, pneumonia, and enteritis, with intralesional gram-negative coccobacilli.^16^ These findings are similar to those observed in our 3 cases, in which liver and spleen were the most affected organs, followed by the kidney, lungs, skeletal muscles, heart, air sacs, and intestines.

Increased susceptibility to avian pseudotuberculosis in zoo animals is linked to factors such as cold weather, poor management, parasitism, and diet,^5,16^ with stress playing a key role by suppressing immune responses. Stressful events such as noise and activity spikes in exhibits often precede outbreaks in primates, artiodactyls, and birds.^2,11^ In our cases, no stressful event was recorded before the outbreak. The 3 birds died in winter and early spring, which were cold, rainy months that likely increased infection susceptibility and bacterial persistence. Stress resulting from maladaptation to such climates may contribute to this pattern in tropical species. However, it is more likely that this trend reflects the tendency of rodent vectors to seek shelter indoors during the cold season.^11^ During the field investigation, we did not identify a probable rodent invasion in the aviary, given that the zoo has strict protocols for rodent control and food storage.

Iron is essential for the survival and growth of many bacterial organisms and is often a limiting factor for bacterial development.^2,11^ Excessive iron storage in birds, mice, and humans is suggested to predispose them to yersiniosis; the excess iron is believed to facilitate the survival and proliferation of *Y. pseudotuberculosis*. Authors of a study in chickens (*Gallus domesticus*) suggested that iron excess facilitated the development of systemic pseudotuberculosis because of increased iron availability. Toucans (*Ramphastidae*), which are prone to developing iron-storage disease, are considered highly susceptible to the development of pseudotuberculosis. Although no correlation has been confirmed between the amount of stainable iron in the liver and the number of lesions, a correlation has been found between the amount of stainable iron and the size of the bacterial lesions in the liver.^4^ The observation that the severity of lesions caused by *Y. pseudotuberculosis* is greater in birds with abundant iron stores could indicate that increased iron availability may influence the presentation, course, and possible susceptibility to disease in birds. However, other factors, including species differences in diet, concurrent parasitism, and seasonal effects on tissue metabolism, also influence the amount and distribution of tissue iron stores in birds, as well as intestinal iron uptake, depending on the species. These factors could account for a change in susceptibility to systemic disease.^4,8,9^

Perl stain detected mild iron accumulation in the liver and spleen in all 3 cases, with some deposits near necrotic foci (Table 1). No clear differences were observed among the cases. Liver and spleen may be more affected given their role in iron storage and metabolism,^13^ although further investigation is necessary.

The source of exposure to *Y. pseudotuberculosis* remains unknown in our study. Laboratory testing of environmental and carrier samples suggested that the bacteria were not present on fomites or on contact animals. A 12-y-old squirrel monkey had died at the zoo because of *Y. pseudotuberculosis* infection in the previous year (2024); however, the monkey was located far from the affected aviary, with no interspecies contact or shared equipment. The hooded pitta—the first of our bird cases that succumbed to *Y. pseudotuberculosis*—had been the bird introduced most recently into the aviary; it died 5 mo after introduction. Therefore, the outbreak may have originated from the introduction of the pathogen into the flock with this animal. The zoo hospital clinicians implemented measures to control the spread of *Y. pseudotuberculosis*, including an 11-mo quarantine period (2025 Mar 20–2026 Feb 20), and no new cases of yersiniosis have been reported in over a year since the outbreak ended. They also stopped bringing birds into the zoo during (or close to) winter, allowing the birds to acclimate to their new surroundings before the cold and rainy season.

Although the in vivo efficacy of antimicrobial therapy against systemic *Y. pseudotuberculosis* infection is unclear, treatment should still be preceded by antimicrobial susceptibility testing to determine whether acquired resistance is present.^7,16^ Despite the stress of treatment and concerns for antimicrobial resistance, prophylactic therapy for the remaining birds during outbreaks may be warranted to minimize flock health impacts. Fecal cultures may be useful for surveillance, although more prolific enteric bacteria may inhibit the growth of *Y. pseudotuberculosis*, rendering false-negative results; a positive culture should be considered significant.^2^

The role of fomites in transmission is poorly understood. *Y. pseudotuberculosis* may lose virulence genes when adapting to soil life, lowering reinfection risk after 9 mo.^11^ Ventilation, sun, and heat may also help control bacteria, as yersiniosis cases are rarely documented in warm months. Strict control measures in zoos are essential to prevent contact with *Yersinia* bacteria.
